# P-1304. Prevalence and Resistance Patterns of Carbapenem-Resistant Enterobacteriaceae in Bangladesh: A Nationwide Laboratory-Based Study

**DOI:** 10.1093/ofid/ofaf695.1492

**Published:** 2026-01-11

**Authors:** Aninda Rahman, Mohammad Julhas Sujan, S M Shahriar Rizvi, Nimesh Poudyal

**Affiliations:** Directorate General of Health Services, Government of Bangladesh., Dhaka, Dhaka, Bangladesh; International Vaccine Institute, dhaka, Dhaka, Bangladesh; Communicable Disease Control, DGHS, MOHFW, dhaka, Dhaka, Bangladesh; International Vaccine Institute, dhaka, Dhaka, Bangladesh

## Abstract

**Background:**

Carbapenem-resistant Enterobacteriaceae (CRE) pose a significant public health threat, particularly in low- and middle-income countries like Bangladesh, where antimicrobial resistance surveillance remains fragmented. CRE infections are major contributors to healthcare-associated infections (HAI) and are associated with high mortality, limited treatment options, and increased healthcare costs. This study evaluates the prevalence, resistance patterns, and temporal trends of CRE in Bangladesh using data from standard public and private microbiology laboratories.

**Figure:**
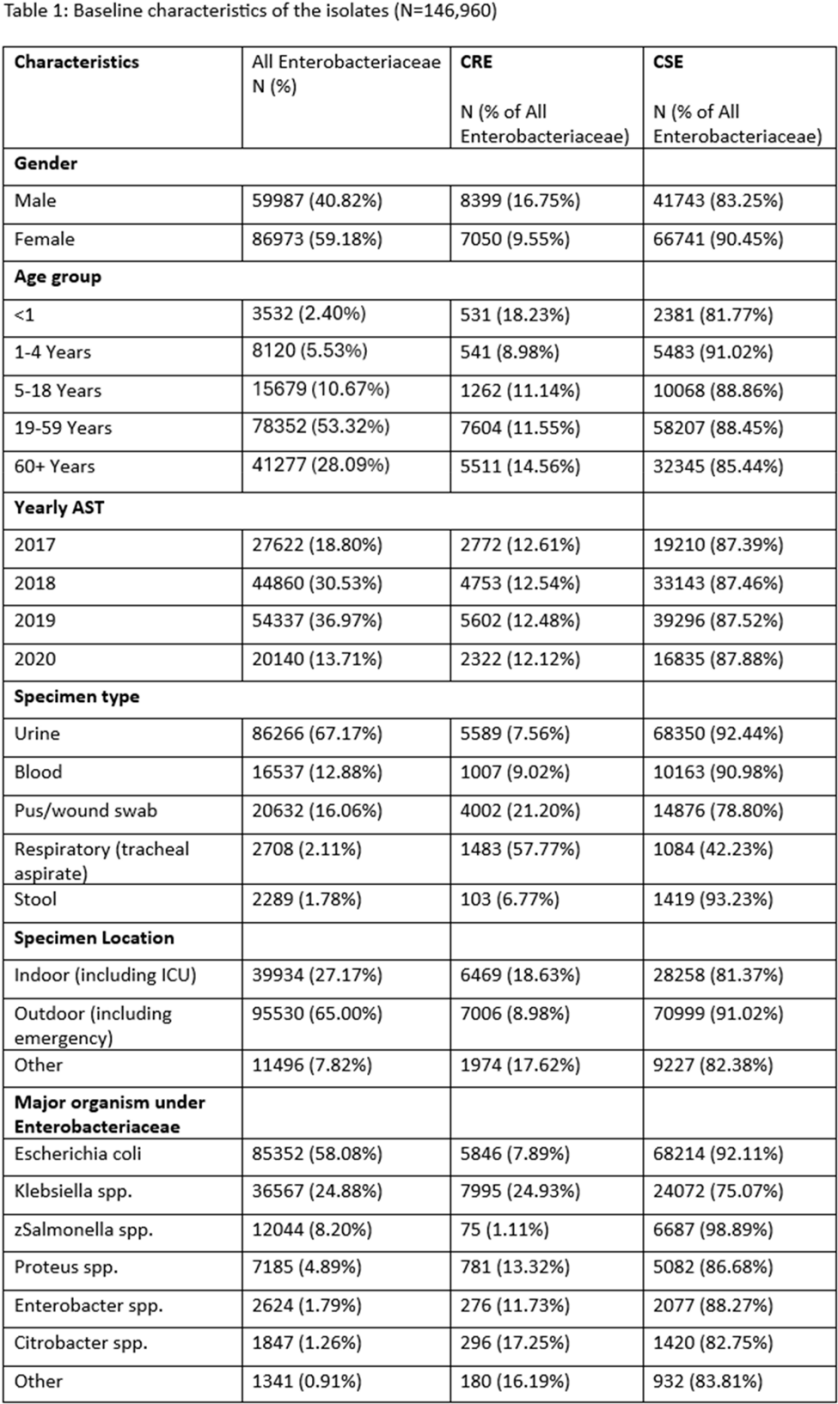

**Figure:**
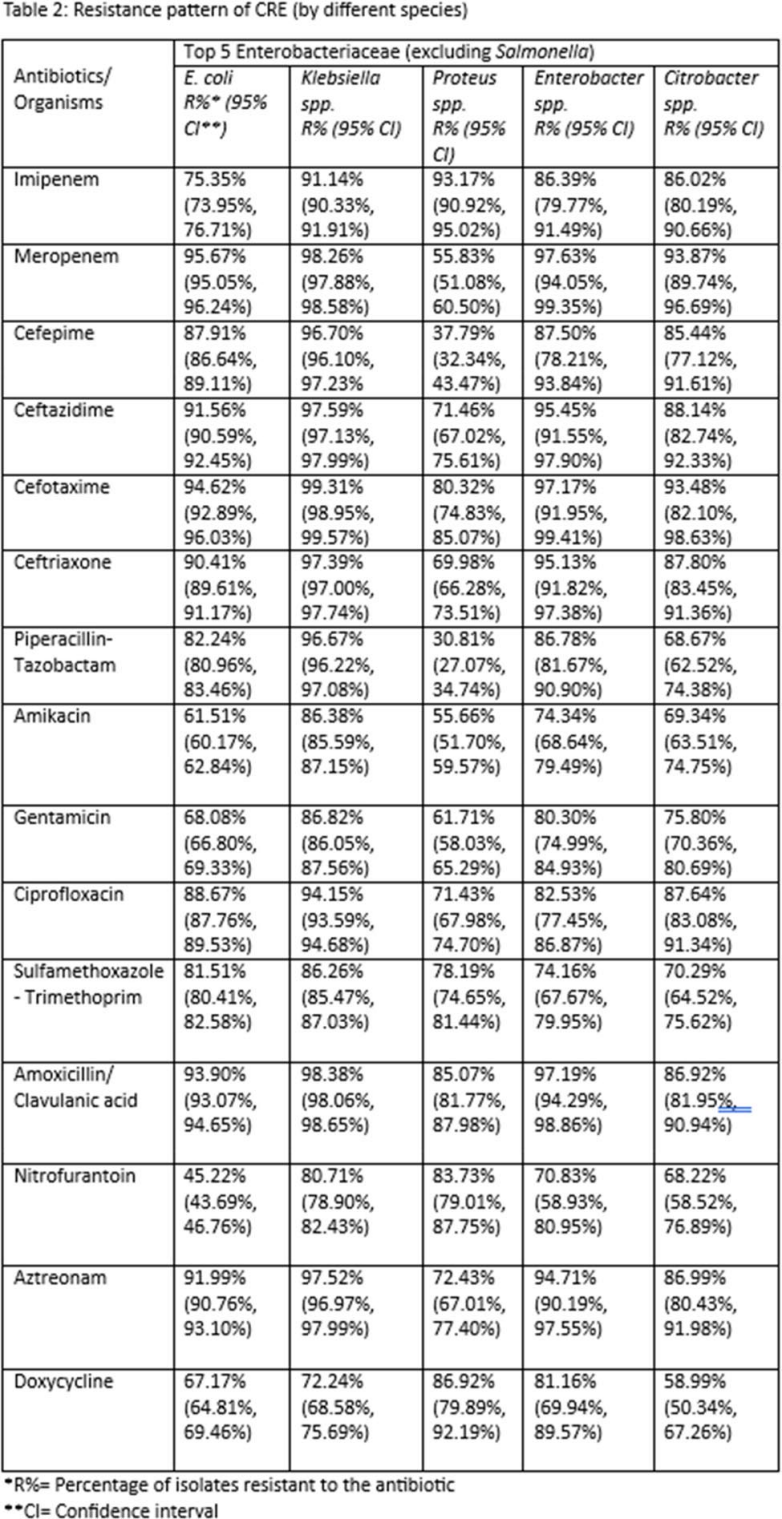

**Methods:**

We conducted a retrospective, cross-sectional analysis of antimicrobial susceptibility testing (AST) data (from 2017 to 2020) from 34 microbiology laboratories across Bangladesh. Before data collection, each laboratory underwent a quality evaluation using a predesigned questionnaire and a physical assessment. CRE was defined based on Clinical & Laboratory Standards Institute (CLSI) guidelines. Data were standardized using WHONET and analyzed using STATA 17.

**Results:**

Of 146,960 Enterobacteriaceae isolates, 12.47% (n=15,449) were identified as CRE. Males (16.75%) were more frequently affected than females (9.55%), with the highest incidence among patients aged ≥60 years (14.56%) and < 1 year (18.23%). The prevalence of CRE increased from 12.12% in 2017 to 12.61% in 2020. CRE was more frequently detected in tracheal aspirates (57.77%) and wound swabs (21.20%) compared to urine (7.56%). Indoor samples showed a higher prevalence (18.63%) compared to outdoor samples (8.98%), suggesting a role of HAI. Klebsiella spp. (24.93%) had the higher proportion of CRE than E. coli (7.89%). All CRE isolates showed a high level of resistance against most antibiotics. Resistance was highest to cefotaxime (94.62%–99.31%), ceftazidime (91.56%–97.59%), and aztreonam (91.99%–97.52%). Resistance to amikacin and gentamicin remained lower at 61.51%–86.38% and 68.08%–86.82%, respectively.

**Conclusion:**

CRE prevalence in Bangladesh remains high, with increasing resistance to carbapenems and third-generation cephalosporins. These findings highlight the urgent need for enhanced infection prevention, antimicrobial stewardship, and routine CRE surveillance to mitigate further spread.

**Disclosures:**

All Authors: No reported disclosures

